# Clinical Outcomes of Endodontic Treatments and Restorations with and without Posts Up to 18 Years

**DOI:** 10.3390/jcm10050908

**Published:** 2021-02-25

**Authors:** Denise Irene Karin Pontoriero, Simone Grandini, Gianrico Spagnuolo, Nicola Discepoli, Stefano Benedicenti, Valerio Maccagnola, Alberto Mosca, Edoardo Ferrari Cagidiaco, Marco Ferrari

**Affiliations:** 1 Department of Prosthodontics and Dental Materials, University of Siena, 53100 Siena, Italy; denisepontoriero@yahoo.it (D.I.K.P.); edoardo.ferrari.cagidiaco@gmail.com (E.F.C.); 2 Department of Endodontics and Restorative Dentistry, School of Dental Medicine, University of Siena, 53100 Siena, Italy; simogr@gmail.com; 3 Department of Neurosciences, Reproductive and Odontostomatological Sciences, University of Naples “Federico II”, 80131 Naples, Italy; gspagnuo@unina.it; 4 Department of Periodontology, School of Dental Medicine, University of Siena, 53100 Siena, Italy; ndiscepoli@me.com; 5 Department of Endodontics and Restorative Dentistry, School of Dental Medicine, University of Genoa, 16121 Genoa, Italy; benedicenti@unige.it; 6 Department of Orthodontics, University of Padua, 35122 Padua, Italy; maccagnolacomunicazioni@gmail.com; 7 Private Practitioner, 25025 Brescia, Italy; amosca@gmail.com

**Keywords:** build-up, clinical trial, endodontic outcomes, endodontic retreatments, posts

## Abstract

Background: The aim of this study was to collect long-term restorative and endodontic outcomes of endodontically treated teeth (ETT). Methods: 298 teeth were included in the study and were recalled up to 18 years with a media of 10.2 years. At baseline, 198 sample teeth (66.44%) showed symptoms and 164 (55%) had periapical radiolucency. The most frequently used obturation techniques were warm gutta-percha in 80% of cases, and by carrier in 20%. A total of 192 ETT were restored by direct resin composite restorations, and 106 posts were luted. Moreover, 75 (25.16%) direct restorations remained as final restorations, 137 single crowns (45.97%), 42 (14.09%) partial adhesive crowns, and 42 (14.09%) abutments of fixed bridges were the final treatments. Descriptive and inferential statistics were performed (*α* = 0.05). A Cox regression model was made. Results: results showed success for 92.6% of ETT up to 18 years, 2.68% (8 ETT) showed irreversible failures, and 14 (4.69%) reversible complications. Four ETT (1.34%) failed because of root fracture and the other four (1.34%) because of endodontic complications. Eight ETT (2.69%) showed non-irreversible periodontal complications and the other six (2.01%) prosthodontic complications. Accordingly, with Kaplan–Meier analysis, the survival rate after 18 years was 97.3% (Interval of Confidence (IC) 95.1–98.3). The presence of a short or long (at least 1 mm related to radiographic apex) quality endodontic filling displayed a statistically significant higher risk of complication (hazard ratio (HR) = 17.00 (IC 5.68–56.84). Furthermore, a clinically detectable not precise coronal margins predicts the presence of any clinical complication with a hazard ratio almost seven times higher than endodontically treated teeth with a proper margin (HR = 6.89 (IC 2.03–23.38)), while the presence of lucency at the baseline did not affect the risk of complication (HR = 0.575 (IC 0.205–1.61)). The presence of post, tooth position in the arch, and the type of it did not show a high-risk rate (HR = 1.85, 1.98, and 2.24, respectively). Conclusions: a correct filling (at the apex) of root canals combined with proper coronal margins allow obtaining a long-term high success rate in teeth with a periapical lesion at the baseline. The use of a post or not, when its placement is related to the residual amount of the crown, does not change the final outcome of the ETT.

## 1. Introduction

Long-term survival of endodontically treated teeth (ETT) depends on correct and well-sealing restoration and the principles of endodontic therapy, by outlining the biology of the dental pulp and periradicular tissues, the etiology and pathophysiology of the disease processes, and the measures to diagnose, prevent, and cure the different disorders that have been established [1,2].

Different parameters were proposed to define endodontic “success” and the existing data on endodontic therapy outcome must be carefully interpreted. Differences in the assessment of teeth during follow-up were noted, such as the radiographic assessment method, the radiographic criteria for success (loose and strict), the unit of outcome measure (root and tooth), and the length of follow-up [3].

Criteria setting the threshold for success at the complete resolution of the periapical radiolucency have been described as “strict” [4] or “stringent” [5], while choosing a mere reduction in the size of the periapical radiolucency [6], has been described as setting a “loose” [4] or “lenient” [5] threshold. The frequency of adoption of these two thresholds in previous studies has been similar; the expected success rates using “strict” criteria would be lower than those based on “loose” criteria. Regarding periapical periodontitis in radiographs, a scoring system for registration and evaluation was proposed [7]. This system provides an ordinal scale of five scores, ranging from healthy to severe periodontitis with exacerbating features. It is based on radiographs with verified histological diagnosis and can be suitable in epidemiological studies.

Another outcome measure, “functional retention”, has been introduced [5]. A root can be considered “functional” when no clinical signs and symptoms are present, independently from the presence or absence of periradicular radiolucency [8,9].

“Functional retention” of teeth after root canal treatment is a similar but less lenient outcome measure than “survival”. 

The “strict criteria” are based on remission of clinical signs and symptoms, and lamina dura’s complete restitutio ad integrum [10] were followed in this clinical study. Considering that the shortest recall in this clinical study was performed at 48 months, there was reasonable time for complete healing of the wide majority of periapical lesions (when present) and, in this way, “strict” criteria were followed. 

Endodontic treated teeth must be restored in order to function and for esthetic purposes. Restoration of ETT can be performed with or without a post [11], by direct or indirect restoration. The best treatment is still under discussion, but it depends on the amount of the residual crown, the anatomy of roots, etc. [12,13,14,15,16].

The type of restoration made on top of the endodontic treated root is another clinical key factor to guarantee longevity to the tooth and, in particular, the precision of the coronal margin of the restoration, independently, if an indirect or direct restoration is made [17,18,19]. Another important aspect is how the endodontic treated root can be built up, using or not posts [20]. It is still an open question whether a post is needed or can be avoided [11,21,22].

In order to obtain predictable results, clinical trials are needed. Clinical trials are considered more reliable than in vitro tests and can be retrospective or prospective [23,24].

Prospective clinical studies are usually performed in specialized centers; specific parameters are evaluated with a limited number of samples. Retrospective studies can collect a wider number of samples and may reflect the clinical behavior of practitioners.

The aim of this retrospective clinical study was to collect long-term restorative and endodontic outcomes of ETT restored by different clinical procedures.

The tested null hypotheses were: (1) there was no difference in the endodontic outcome of ETT with and without periapical lesion at the beginning of the treatment; (2) there was no difference in the endodontic outcome between endodontic treatments with or without precise coronal margins; (3) there was no difference between ETT restored with or without post; (4) there was no difference between ETT restored by direct or indirect restoration.

## 2. Materials and Methods

### 2.1. Study Population

Over a 20-year period (March 1999 to March 2019), one expert endodontist (DP) conducted 298 endodontic treatments in 205 patients (143 men, age range: 19 to 61 years; 169 women, age range: 19 to 71 years); follow-ups were done with reference to the dental records. Patients were in need of different endodontic therapies. Consecutive patients were selected from the authors’ offices. Only primary endodontic treated teeth or nonsurgical retreatments, with a follow-up of at least 18 months or longer, were included in this survey, with patients who returned for oral hygiene recalls in 2019.

All procedures performed in this study involving human participants, were in accordance with the ethical standards of the institutional committee, and with the 1964 Helsinki declaration and its later amendments or comparable ethical standards. Informed written consent was obtained from all individual participants included in the study. Collection and analysis of the data were approved by the Ethical Committee of the University of Siena. 

Inclusion criteria were the following: age: 42 (±7.9) years (range 19 to 71); sex: 169 F, 143 M; periodontally healthy or successfully treated patients in need of one or more endodontic treatments.

Exclusion criteria were the following: individuals who were not yet adults (<18 years), pregnancy, disabilities, previous prosthodontic restorations of abutment teeth, deep defects (close to pulp, <1 mm distance), or pulp capping, heavy occlusal contacts or history of bruxism, systemic disease or severe medical complications, allergic history concerning methacrylates, rampant caries, xerostomia, lack of compliance.

### 2.2. Sample Characteristics

A total of 298 teeth were included in the study, of which, 52 were premolars (26%), 170 molars (52%), and 76 anterior teeth (21%, II e V sextant); 103 ETT belonged to the mandible (30.68%) and 195 (69.32%) to maxillae. 

Several endodontic peculiarities were analyzed. At baseline, 198 sample teeth (66.44%) showed symptoms (tenderness/pain to percussion) and 164 (55%) had periapical radiolucency. Regarding ETT with radiolucency, 90 of them (54.87%) were in coincidence with their need for retreatment and 74 (45.13%) were necrotic teeth. The most frequently used obturation techniques were warm gutta-percha in 80% of cases, by carrier in 20%, and hot gutta-percha in other cases, mainly when curve canals were treated.

After being endodontically treated, 192 ETT were restored by direct resin composite restorations, and 106 posts were luted. A total of 75 (25.16%) direct restorations remained as final restoration, 137 single crowns (45.97%), 42 (14.09%) partial adhesive crowns, and 42 (14.09%) abutments of fixed bridges were the final treatments.

### 2.3. Original Endodontic Therapy Procedure

For each tooth, the following preoperative data were recorded: demographic data, tooth location, number of root canals, previous endodontic treatment, clinical signs and symptoms, vitality tests, and radiographic periapical status. Based on these findings, the preoperative condition was classified as one of the following: vital (healthy or irreversibly inflamed pulpitis), non-vital, endodontically treated, with or without periapical lesion, and symptomatic or asymptomatic. 

For each tooth, the following intra-operative data were recorded: number of treatment sessions; inter-appointment dressing (if used); the occurrence of procedural complications such as perforation, breakage of files and flare-up; length of canal filling (at apical level, 1 mm short or more and beyond); and temporary restoration placed. A conservative endodontic cavity (CEC) access was performed using a long shaft round diamond bur and endodontic dedicated ultrasonic tips. After straight-line access preparation was obtained, root canals were negotiated with pre-curved stainless steel K-type files (Maillefer, Bailague, Switzerland), size 0.8 or 10 ISO (International Standard Organization) to the major apical foramen. Working length was measured using an electronic apex locator (Root ZX Morita, Tokyo, Japan), established at electronic 0 and, in most cases, checked with an intraoperative X-ray. Due to the long period of time that has been taken into consideration in this study, different shaping techniques and instruments have been used. From 1999 to 2003 a crown-down approach was utilized to give correct shaping to the canals; pre-flaring was performed with a manual pre-curved stainless steel K-file with a #25 tip 0.02 taper, then the shaping was performed with a Ni-Ti rotary file system, which had tip size #25 for all instruments and a different taper (QANTEC Kerr, Kerrville, TX, USA From 2003 to 2013, a simultaneous technique was introduced in the clinical procedure, utilizing Ni-Ti rotary files with different tip sizes and different tapers (Mtwo, Sweden e Martina, Italy). From 2013 to 2019, a mixed technique was adopted: pre-flaring and glide path were performed to length with a nickel-titanium #10 tip size and 0.04 taper rotary file, followed by a nickel-titanium #15 tip size and 0.05 taper rotary file (Mtwo, Sweden e Martina, Italy). All canals were shaped with the M-Wire alloy rotary instrument ProTaper Next (Maillefer, Bailague, Switzerland) to a length of up to a #25 tip size and a variable taper. The apical diameter was measured (apical gauging) using nickel-titanium manual K-type files, NiTi Flex (Maillefer, Bailague, Switzerland), and the shaping of the apical third was refined, where needed. Irrigation was copious and frequent using heated 5.25% sodium hypochlorite NiClor (NiClor, Ogna, Bologna, Italy) deposited with side-vented 30-G needles. After instrumentation, the root canals were irrigated with 17% EDTA solution Tubuliniclean (Ogna, Bologna, Italy), for 3 min, followed again by several 1-min irrigations with heated 5.25% sodium hypochlorite solution. 

The canals were dried with dedicated sterile paper points, filled with dedicated gutta-percha cones ProTaper Next (Maillefer, Bailague, Switzerland), and zinc oxide-based endodontic sealer (Pulp Canal Sealer, Kerr, Germany) using a continuous wave of condensation technique (80%) or a carrier-based technique (Thermafil, Dentsply, Konstanz, Germany) in roots with curve canals, depending on the root canal anatomy. A post was placed when the remaining coronal structure was less than 50% [25]. A temporary restoration was performed using zinc oxide based cement placed on the pulp chamber floor covered by a layer of glass ionomer cement (GCem, GC Co., Tokyo, Japan).

The post space was prepared using the drill provided by the manufacturer. Fiber-reinforced composite post was adapted to the anatomy of the root. Post length was adapted to the length of the post space. The post surface was cleaned with phosphoric acid and treated with a silane-coupling agent. For adhesive cementation, the dentinal surface was etched with phosphoric acid for 10 s and pretreated with a dual-cure adhesive before the post was cemented with a dual-cure resin. Aesthetic Plus fiber posts in combination with the All Bond 2 bonding system and proprietary C&B resin cement (Bisco) were used between 1999 and 2008. GC fiber posts, in combination with Gradia Core (GC), were used from 2009 to 2018. Porcelain to fused metal crowns were cemented with Fuji Cem (GC) until 2015, whilst more recently, zirconia full crowns were luted with G-Cem adhesive cement (GC). When direct restorations were placed, cuspal coverage was made, and the restorations were made using resin composite materials in combination with proprietary bonding systems. From 1999 to 2010, Gradia (GC) resin composite in combination with G-Bond (GC) was used. After 2010, a combination between G-aenial resin composite (GC) and G-Bond Plus (GC) was used. More recently (from 2016), the same resin was used in combination with GPremio bond (GC).

### 2.4. Follow-Up

For each tooth, the following postoperative data were recorded: the treatment and recall period, the presence or absence of signs and symptoms, the presence or absence of apical lesion, the presence and type of restoration, and the type of build-up with or without a post. Only primary endodontic treated teeth or nonsurgical retreatments with a follow-up of at least 18 months or longer were included in this survey with a media of 10.2 years. The follow-up sessions were performed with patients who returned to the offices during oral hygiene recalls during 2019. Among all patients who returned for a recall, 298 teeth were selected for this survey. All of the recorded information from the files were transferred to a computerized database. The clinical follow-up examinations were performed by the primary author (D.P.). For teeth examined more than once, only the findings of the final examination during 2019 were considered. Traumatized teeth, injured with luxation, intrusion, extrusion, avulsion, or horizontal fractures, and teeth requiring endodontic surgery, were excluded from this study. 

### 2.5. Criteria of Evaluation

When only the endodontic treatments were evaluated, the following criteria of the European Society of Endodontology 1994 [25] were used to judge the success rate of root canal therapy: (1) clinical examination: the absence of pain, swelling, and other symptoms, no sinus tract, and no loss of function; and (2) radiographic examination: the periodontal ligament space was normal on the original diagnostic radiograph, and it remained unchanged on recall radiographs, or healing of a radiolucent area visible on the original preoperative radiograph was observed and the periodontal ligament space returned to normal. For radiographic examination, PAI (Peri Apical Index) scores were used [7].

Therefore, cases were considered as failures in the presence of pain, swelling, and sinus tract. Radiographically, failures were identified when a lesion appeared after endodontic treatment, when a preexisting lesion increased in size, and when a lesion remained the same or only diminished in size. Multi-rooted teeth were assessed according to the root that appeared the worst. 

Debonding of the post was registered when the crown dislodged or/and moved. Loss of retention was registered when mobility was detected between the crown and the abutment, when saliva was expressed at the margin of the crown when pressure was applied, or when an explorer could easily be inserted between the tooth and the crown. 

Coronal fracture of the direct restoration was registered when visible. 

Carious lesions were recorded when a dental explorer could penetrate the dentin at the cervical margin of the crown or the direct restoration as assessed through radiographs and/or clinically. 

Ceramic fractures (chipping) were registered clinically and from photographs. 

Possible “marginal leakage” was clinically evaluated with a sharp explorer along the margins and radiographically.

Periodontal involvement was recorded when a periodontal disease (not existing at the baseline) was visible around the sample tooth according to periodontal parameters [26].

The two examining operators (A.M. and V.M.) were calibrated before the examination. Calibration was done in 30 cases. The two examiners made their own evaluations. In case they disagreed, the single cases were reevaluated and an agreement was found.

In this clinical study, the endodontic and restorative treatments made for each tooth were evaluated in a single sample. For that, when the global treatments were considered, it was decided to avoid misunderstanding between the definitions of success and survival. Success was defined by the percentage of endodontic treatments and restorations that remained in situ without any modification. Survival was defined by the percentage of endodontic treatments and restorations that remained in situ, with modifications, but still under clinical acceptability. Failure was defined by the percentage of teeth that needed to be replaced [27].

Data were collected based on predetermined criteria. Percentages of teeth with or without apical periodontitis were recorded, as well as adequate root canal treatment (AE), inadequate root canal treatment (IE), adequate seal of the restoration (AR), and inadequate seal of the restoration (IR). 

### 2.6. Radiographic Method and Evaluation

When evaluating treatment results, the first clinical and radiographic examination was performed by the primary author (D.P.) when the 298 followed cases were randomly selected from patient files, recorded by handwriting, and the recorded information was transferred to a computerized database. The final evaluation was done together with two other observers after calibration (A.M. and V.M.). For radiographic examination, PAI scores were used [7].

### 2.7. Statistical Analysis

Descriptive and inferential statistics were performed using the Stata 15 IC statistical package, and the significance level was set at *α* = 0.05. The minimum, mean, and maximum follow-ups were calculated for all endodontic treatments. The overall failure incidence rate was according to the total failure events (tooth extraction) and to the presence of any complication (free of the event) divided by the total tooth-years during the total follow-up period; the 95% confidence intervals (95% CI) were estimated according to the Poisson distribution. Both the cumulative survivals performed were recorded using the Kaplan–Meier analysis. Cox regression analysis was used to examine the risk factors for any clinical event during the follow-up period. The results were presented as hazard ratio (HR) with 95% confidence intervals (CI). 

## 3. Results

**Figure 1:**
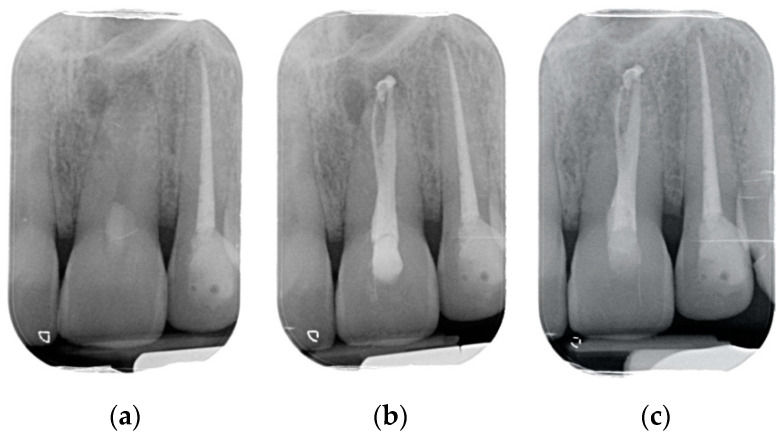
(**a**) Upper central incisor with periapical lesion. (**b**) The root after being endodontically treated. A second root was filled by warm gutta-percha technique. A direct resin composite restoration was used to restore the coronal part of the tooth. (**c**) The root after 3 years of clinical service. A complete healing is evident.

**Figure 2:**
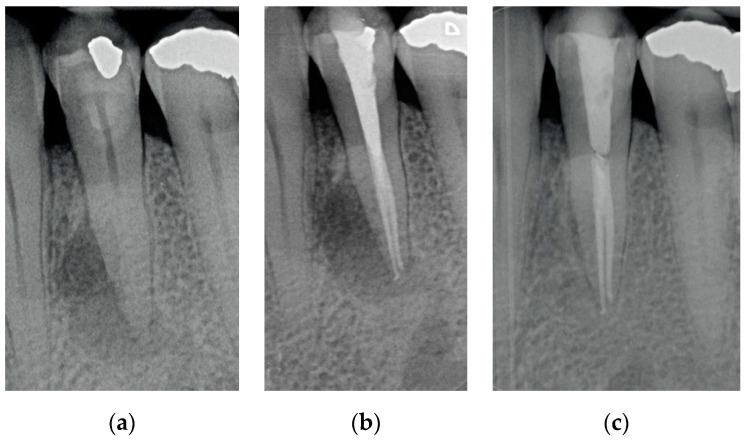
(**a**) A lower bicuspid in need to be endodontically treated because of necrosis of the pulp and periapical lesion; (**b**) The tooth after being endodontically treated. Two canals were found, cleaned and filled; (**c**) The tooth at 2 years recall. The periapical lesion completely disappeared.

**Figure 3:**
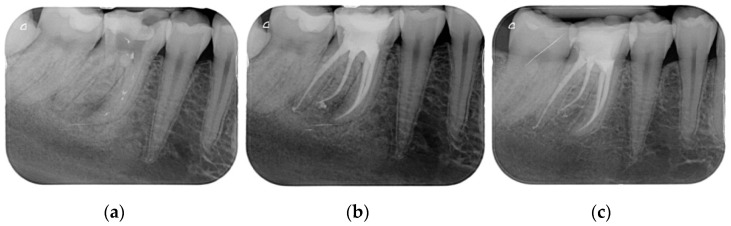
(**a**) A first lower molar with periapical lesion and in need to be retreated. (**b**) The molar immediately after being endodontically treated. Four canals (one in a radix entomolaris) were detected and treated. (**c**) The molar after 4 years. The good health of the periapical areas can be noted. The crown was restored with an adhesive esthetic onlay.

Results showed that 92.61% of ETT did not have any complications up to 18 years (Figure 1, Figure 2 and Figure 3).

Regarding the quality of root filling, 264 teeth (88.59%) showed good filling (Figure 1, Figure 2 and Figure 3) (gutta-percha at the radiologic apex), 22 roots (7.38%) short filling (shorter than 1 mm from the radiographic apex), and 12 (4.03%) long filling (longer than 1 mm of the radiographic apex).

There were no statistically significant differences among outcomes of vital root and necrotic and retreatment with radiolucency.

At the recall, 284 (94.42%) ETT showed good coronal margin, 11 (4.38%) not-so good margins (margin was filled non-precise with the sharp explorer), and 3 (1.2%) clear opening of the margins radiographically.

Success was recorded in 276 ETT (92.6%), 2.68% (8 ETT) showed irreversible failures, and 14 (4.69%) reversible complications.

Four ETT (1.34%) failed because of root fracture and the other four (1.34%) because of endodontic complications.

Eight ETT (2.69%) showed non-irreversible periodontal complications and the other six (2.01%) prosthodontic complications.

The placement or not of a post did not show different outcomes.

Sixteen failures were found when a full crown was cemented on the ETT. Failures were recorded in three cases in combination with direct restorations, in one case with a partial crown, and two cases as the abutment of a bridge.

**Figure 4:**
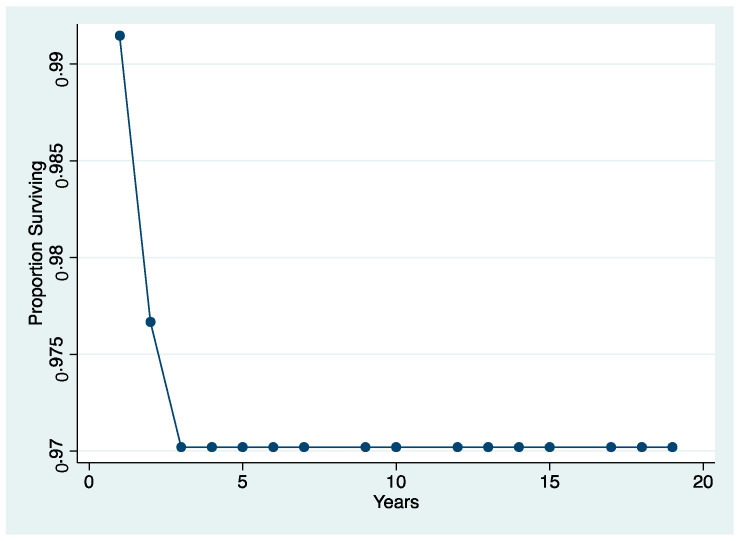
The survival rate after 18 years was 97.3% (Interval of Confidence (IC) 95.1–98.3). All of the extractions, except, one occurred during the first 2 years of service.

**Figure 5:**
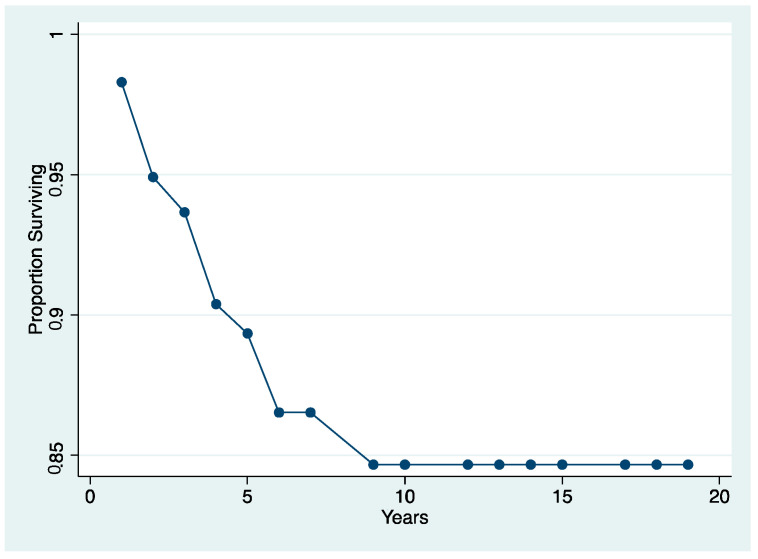
The cumulative survival to any complication displays that they occurred in the vast majority during the first 5 years of service. Notwithstanding, after 18 years of observation, the success rate was 92.6% (IC 90.1–94.71).

The survival rate of both “failure” and “free of the event” of endodontically treated teeth, determined by the Kaplan–Meier analysis over an 18-year period, is shown in Figure 4 and Figure 5. The survival rate after 18 years was 97.3% (Interval of Confidence (IC) 95.1–98.3). All of the extractions, except one, occurred during the first 2 years of service. The cumulative survival to any complication displays that they occurred in the vast majority during the first 5 years of service. Notwithstanding after 18 years of observation, the success rate was 92.6% (IC 90.1–94.71).

### Cox Regression Analysis

A Cox regression model was built to verify the predictive potential for survival data to any complication of clinical and radiographic variables. The independent variables were evaluated in terms of the hazard ratio (Table 1). The final model obtained by the command “all sets” (Stata 15 IC displays a risk of any complication five times higher for the presence of a full crown as a final restoration (HR = 5.03 (IC 1.39–18.20)) in comparison to any other restorative procedure. The presence of a non-perfect quality of the endodontic filling (short or long) displayed a statistically significant higher risk of complication (HR = 17.00 (IC 5.68–56.84). Furthermore, clinically detectable non-precise margins predict the presence of any clinical complication with a hazard ratio almost 7 times higher than endodontically treated teeth with proper coronal margins (HR = 6.89 (IC 2.03–23.38), while the presence of lucency at the baseline did not affect the risk of complication (HR = 0.575 (IC 0.205–1.61). The presence of post, tooth position in the arch, and type did not show high-risk rate (HR = 1.85, 1.98, and 2.24, respectively). 

## 4. Discussion

The long-term survival and success rates of ETT are similar and/or better than those of implants available in the literature [28,29,30]. For that, it is mandatory to safe natural teeth as many as possible. High success and survival rates of ETT are mainly related to the quality of the endodontic treatment and the restorative procedure used to save the tooth in clinical services [31].

In this clinical study, many parameters were collected and statistically evaluated on a long-term basis. The clinical samples were followed up to 18 years and failures were observed mainly within the first years of clinical service. This finding showed that when root fracture was avoided by covering cusps with the crown or the direct restoration, the teeth were protected from occlusal loading [32]. Moreover, when partial or complete healing of the periapical lesion was achieved, the restored root remained in clinical service without any clinical sign or symptom. These findings were in agreement with previous reports [8,33,34,35]. 

The presence of signs and symptoms—including the presence of periapical lucency—did not influence the final outcomes. In fact, around half of ETT showed a radiolucency visible at the baseline, and of them, approximately 50% were present in teeth in need of retreatment, whilst the others were necrotic teeth. No differences were found in the final outcomes among teeth with radiolucency at the baseline (necrotic and roots in need of retreatment) and those without (vital teeth). For that, the first null hypothesis—that there was no difference in the endodontic outcome of ETT, with or without periapical lesions at the beginning of the treatment—was accepted

The numbers of failures due to tooth fractures, endodontic, periodontal, or prosthodontic reasons, were limited to 22 of 298 ETT. Of the recorded failures, 14 were reported as repairable; eight were catastrophic failures and, consequently, needed root extraction. The success rate was around 92% (Figure 1, Figure 2 and Figure 3), the survival rate around 4.69%, and only less than 2.69% were irreversible failures. The success and survival rates of this clinical study were a little higher than several others [36,37,38]. Another important aspect related to the failure was the fact that irreversible failures mainly took over in the first two years and within the first 5 years, when cumulated as reversible and irreversible failures. It can be speculated that “biological” complications can come out rather quickly, and periodontal and prosthodontic complications in a longer time, but after 5 years of clinical service, it can be expected that an ETT can stay in clinical service for many more years. 

One-third of the restored ETT were in the mandible and two-thirds in the maxilla, but no statistically significant differences were found in the outcome. 

The endodontic standardized procedures used in this study were strictly followed, which could be considered other important factors that determine high-quality outcomes. At the baseline (immediately after endodontic treatment was completed) a precise root filling at the radiographic apex of root canals was recorded (approximately 92%), while in less than 5% the root filling was short, and in less than 4% too long. The quality of endodontic treatment, and in particular of root filling, could be an important factor used to predict a positive outcome [17,18,19]. 

From the result of this clinical study, there was no difference between final outcomes of vital teeth and second root canal treatments. It was expected that the presence of periapical translucency, teeth already endodontically treated, and/or necrotic teeth can determine lower success and survival rates. These data can be related to the high-quality root canal fillings and bacteria-tight post-endodontic restorations that were made in this clinical study [39,40].

Regarding the survival and success rates of the restoration made on ETT, several aspects can be pointed out. First, the presence (or not) of the post did not make statistically significant differences. For that, the null hypothesis—that there was no difference between ETT restored with or without a post—was accepted. This might be because posts were placed when clinically indicated. This study is in agreement with other authors [20]. 

The type of restoration was evaluated in relation to success, survival, and failure rates. Statistically, there were no differences between the different types of restorations. 

However, accordingly, with Cox regression analysis evaluating the high-risk ratio, the placement of a crown on the ETT raises the risk of failure, five times more than any other type of restoration. This could partially be because nearly 75% of ETT were restored with a full or partial crown, or abutment of the bridge, raising the number of possible failures combined with crowns. A wider number of samples should be evaluated in order to confirm these findings.

It should note that, in this study, the clinician performing the work was an expert endodontist. The variable “operator” could be considered one of the most important factors concerning the outcomes in dentistry [41]. Experience, knowledge, and skill of the operator can justify the high rate of success and survival for up to 18 years.

When risk ratio analysis was performed, restoration with a proper quality of obturation, and a good marginal coronal seal, were significant factors to obtain long-term high-quality outcomes. These findings are in agreement with several other studies [17,18,19].

For that, the null hypothesis—that there was no difference in endodontic outcome between endodontic treatments, with or without precise coronal margins—was rejected.

Several limitations of this study can be pointed out. First, the limited number of ETT should be expanded; moreover, the outcomes are mainly related to the skill and knowledge of one expert operator, and it would be interesting to enlarge the number of operators. 

The study findings should be confirmed by other (similar) multi-center clinical trials, possibly prospectively made. 

## 5. Conclusions

From the findings of this clinical study, the following conclusion can be drawn: a correct filling (three-dimensional obturation) of root canals, which is the final result of a proper treatment protocol, combined with a good coronal marginal seal, allows obtaining a long-term high success rate in teeth with a periapical lesion at the baseline. 

The presence of a periapical lesion at the baseline does not decrease the quality of the final outcome.

## Figures and Tables

**Table 1:** Considered independent variables. (FullC = full crown; FilQuality = quality of endodontic filling; Symt = presence of symptoms; Seal = quality of restoration margins).

_t	Hazard Ratio	Std. Err.	*z*	*P* > |*z*|	(95% Conf. Interval)
Post	1.444597	1.288705	0.41	0.680	0.251421 8.300259
FullC	2.361482	2.034491	1.00	0.319	0.4363625 12.77973
FilQuality	11.0516	8.10662	3.28	0.001	2.624511 46.53742
Symt	0.7417658	0.5672379	−0.39	0.696	0.1657075 3.320409
Seal	4.425534	4.166967	1.58	0.014	0.6990313 28.01785
